# Agenda

**DOI:** 10.1080/13814788.2016.1277093

**Published:** 2017-03-29

## 2017

**Table:** 

March	
**30-31, Dublin, Ireland**. ***EPCCS****(European Primary Care Cardiovascular Society)* http://www.epccs.eu/d/516/epccs-9th-annual-scientific-meeting	The EPCCS annual meeting is an educational opportunity for those working in primary care with an interest in cardiovascular disease, the single most important disease presenting to primary care physicians.
**30-01 (April), Paris, France.** ***CMG****(Collège of Médicine Générale)* http://www.congresmg.fr/index.php/en/	The Congress of general practice France (CMGF) has been created by and for GPs. It gathers in the same place all the major players in the field: • Specialists of general practice (students, interns, young doctors, teacher-researcher, university training supervisors, practising family physician), • Experts from other medical disciplines, • Representatives from health and research institutions.

**Table:** 

May	
**11-14, Riga, Latvia**. ***EGPRN Spring meeting****(European General Practice Research Network)* http://www.egprn.org	**Theme: Reducing of risk of non-communicable diseases in General practice/Family medicine** Topics: Screening program; Prevention of non-communicable diseases; Life style behavioural changes; Personal resources
**25-28, Budva, Montenegro**. ***AGP/FM SEE****(Association of General Practice/Family Medicine of South-East Europe)* http://www.porodicnamedicina.org.me/en/agpfmsee-2/	**Theme: Healthier in SEE, what can we do?** Topics: Improvement of quality care in gp/fm; Mass non-communicable diseases; Rational use of antibiotics and gp/fm Ethics in family medicine; Workers health and family medicine; Open topics

**Table:** 

JUNE	
**28-01 (July), Prague, Czech Republic**. ***Wonca Europe*** http://www.woncaeurope2017.eu	**Theme: General Practice in Europe: growing together in diversity** ‘United in Diversity’ is the official motto of the European Union. Having been already united in WONCA family we want to grow as a discipline in European diversity by learning from each other and exchanging experience and knowledge. This explains a theme of our Conference: ‘Growing together in Diversity.’ It allows us to open all dimensions of the discipline.

**Table:** 

October	
**19-22, Dublin, Ireland**. ***EGPRN Autumn meeting****(European General Practice Research Network* http://www.egprn.org	**Theme:**

## 2018

**Table:** 

May	
**24-27, Krakow, Poland.** ***Wonca Europe*** http://www.woncaeurope2018.eu	**Theme: Quality, Efficiency, Equity**

**Table:** 

June	
**30 (May)-02, Porto, Portugal.** ***9**th** IPCRG World conference*** http://www.theipcrg.org	**Theme:**

## 2019

**Table:** 

JUNE	
**26-29, Bratislava, Slovak Republic** ***Wonca Europe*** www.woncaeurope2019.eu	**Theme: Quality, Efficiency, Equity**

